# Book Review: Cohn, S.L. (Ed.). Decision Making in Perioperative Medicine: Clinical Pearls. (New York: McGraw-Hill), 2021. ISBN: 978-1-260-46810-6

**DOI:** 10.3390/healthcare9060687

**Published:** 2021-06-07

**Authors:** Richard H. Parrish

**Affiliations:** Department of Biomedical Sciences, Mercer University School of Medicine, Columbus, GA 31902, USA; parrish_rh@mercer.edu; Tel.: +1-(706)-321-7218

Cohn’s work fills a void in the perioperative care literature by providing a concise, comprehensive, practical, and authoritative guide to the medical management of common periprocedural issues and scenarios. The book is organized logically according to the typical flow of patient management, beginning with an introduction to perioperative patient care, prevention of common complications, treatment of co-existing diseases and special populations (such as the cancer patient and surgery in the older patient), and finishing with the management of common post-operative problems.

The book has a number of strengths, and could be easily converted into a computer app to aid utilization for practice enhancement and teaching at the bedside. It contains many helpful tables and figures (algorithms) that assist the reader to form a complete picture of the topic. The importance of conducting a careful risk assessment to avoid exacerbation of co-morbidities or prevention of various post-operative complications is well highlighted. The ‘clinical pearls’ found at the end of each chapter provide the reader with high yield and pertinent information on important treatment decision making aspects. The chapters in the section on common post-operative problems are well-written and succinct.

There are also a number of areas where the book could be improved, perhaps in subsequent editions. Because there are 26 co-authors of the various chapters, the scope, depth, and detail of each chapter seems to vary considerably. Some chapters have guideline or clinical trial citations; some important statements were uncited. For example, in the prevention of surgical site infections (SSI) with antimicrobials, the current workhorse antibiotic and dosing for prophylaxis of SSIs, cefazolin, is not mentioned by name; not all cephalosporins are indicated for prophylaxis [1]. This variation is also true of the ‘clinical pearls’ bullet points, and the source or attribution of the pearl often varies. Some of the pearls are summative statements discussed in-depth in the chapter; others are based on the author’s experience or expertise. Moreover, several citations are not the most up to date, such as the management of post-operative nausea and vomiting (PONV) [2]. In this regard, while PONV is a major reason for delayed discharge from the hospital or outpatient surgery center, its assessment and management are scattered among several chapters. It would have been more helpful to the reader if PONV had its own chapter, placed either in the prophylaxis or common problems section. There are several redundancies and omissions regarding medication management. For example, both the chapter on medication management and ischemic heart disease identify cardiac medications that need to be withheld or continued during the perioperative period. Medications to treat myasthenia gravis (pyridostigmine and azathioprine) are omitted in the robust table (chapter 4, page 34), and importance of avoiding general anesthesia and neostigmine reversal in these patients is paramount to safe emergence by using newer agents, perhaps sugammadex (which is not mentioned in the text at all) [3,4,5]. At times, use of medication names, either brand or generic, is not consistent. There is no chapter on the pediatric patient, which would be another excellent addition to the next edition placed in the special populations section. Considering the recent major paradigm shift in perioperative care culture, the treatment of enhanced recovery programs is somewhat cursory [6,7,8].

On balance, while the book has some minor limitations, it is an excellent collection of tips and wisdom proven to be very helpful in the management of the perioperative patient. It is recommended as required reading for any surgery, general medicine, or pharmacy resident or fellow in training or on rotation, as well as a comprehensive reference for experienced practitioners and allied health professionals working in the periprocedural space. It might also serve as the basis of a shared mental model for collaborative practice development among surgeons, anesthesiologists, hospitalists, clinical pharmacists, nurses, dietitians, and other therapists managing the care of the periprocedural patient.

## Data Availability

Not applicable.

## References

[1] Bratzler D.W., Dellinger E.P., Olsen K.M., Perl T.M., Auwaerter P.G., Bolon M.K., Fish D.N., Napolitano L.M., Sawyer R.G., Slain D. (2013). Clinical practice guidelines for antimicrobial prophylaxis in surgery. Am. J. Health Syst. Pharm..

[2] Gan T.J., Belani K.G., Bergese S., Chung F., Diemunsch P., Habib A.S., Jin Z., Kovac A.L., Meyer T.A., Urman R.D. (2020). Fourth Consensus Guidelines for the Management of Postoperative Nausea and Vomiting. Anesth. Analg..

[3] Sanders D.B., Wolfe G.I., Narayanaswami P., MGFA Task Force on MG Treatment Guidance (2018). Developing treatment guidelines for myasthenia gravis. Ann. N. Y. Acad. Sci..

[4] Collins S., Roberts H., Hewer I. (2020). Anesthesia and Perioperative Considerations for Patients With Myasthenia Gravis. AANA J..

[5] Fernandes H.D.S., Ximenes J.L.S., Nunes D.I., Ashmawi H.A., Vieira J.E. (2019). Failure of reversion of neuromuscular block with sugammadex in patient with myasthenia gravis: Case report and brief review of literature. BMC Anesthesiol..

[6] Smith T.W.J., Wang X., Singer M.A., Godellas C.V., Vaince F.T. (2020). Enhanced recovery after surgery: A clinical review of implementation across multiple surgical subspecialties. Am. J. Surg..

[7] Arrick L., Mayson K., Hong T., Warnock G. (2019). Enhanced recovery after surgery in colorectal surgery: Impact of protocol adherence on patient outcomes. J. Clin. Anesth..

[8] Currie A., Soop M., Demartines N., Fearon K., Kennedy R., Ljungqvist O. (2019). Enhanced Recovery After Surgery Interactive Audit System: 10 Years’ Experience with an International Web-Based Clinical and Research Perioperative Care Database. Clin. Colon. Rectal. Surg..

